# Development and Validation of a Nomogram for Preoperative Prediction of Lymph Node Metastasis in Lung Adenocarcinoma Based on Radiomics Signature and Deep Learning Signature

**DOI:** 10.3389/fonc.2021.585942

**Published:** 2021-04-22

**Authors:** Jia Ran, Ran Cao, Jiumei Cai, Tao Yu, Dan Zhao, Zhongliang Wang

**Affiliations:** ^1^ Engineering Research Center of Molecular & Neuro-imaging, Ministry of Education, School of Life Science and Technology, Xidian University, Xi’an, China; ^2^ Department of Medical Imaging, Cancer Hospital of China Medical University, Shenyang, China; ^3^ Department of Medical Imaging, Liaoning Cancer Hospital & Institute, Shenyang, China

**Keywords:** lung adenocarcinoma, lymph node metastasis, radiomics, deep learning, prediction

## Abstract

**Background and Purpose:**

The preoperative LN (lymph node) status of patients with LUAD (lung adenocarcinoma) is a key factor for determining if systemic nodal dissection is required, which is usually confirmed after surgery. This study aimed to develop and validate a nomogram for preoperative prediction of LN metastasis in LUAD based on a radiomics signature and deep learning signature.

**Materials and Methods:**

This retrospective study included a training cohort of 200 patients, an internal validation cohort of 40 patients, and an external validation cohort of 60 patients. Radiomics features were extracted from conventional CT (computed tomography) images. T-test and Extra-trees were performed for feature selection, and the selected features were combined using logistic regression to build the radiomics signature. The features and weights of the last fully connected layer of a CNN (convolutional neural network) were combined to obtain a deep learning signature. By incorporating clinical risk factors, the prediction model was developed using a multivariable logistic regression analysis, based on which the nomogram was developed. The calibration, discrimination and clinical values of the nomogram were evaluated.

**Results:**

Multivariate logistic regression analysis showed that the radiomics signature, deep learning signature, and CT-reported LN status were independent predictors. The prediction model developed by all the independent predictors showed good discrimination (C-index, 0.820; 95% CI, 0.762 to 0.879) and calibration (Hosmer-Lemeshow test, *P*=0.193) capabilities for the training cohort. Additionally, the model achieved satisfactory discrimination (C-index, 0.861; 95% CI, 0.769 to 0.954) and calibration (Hosmer-Lemeshow test, *P*=0.775) when applied to the external validation cohort. An analysis of the decision curve showed that the nomogram had potential for clinical application.

**Conclusions:**

This study presents a prediction model based on radiomics signature, deep learning signature, and CT-reported LN status that can be used to predict preoperative LN metastasis in patients with LUAD.

## Introduction

Lung cancer is the most common cancer worldwide and the leading cause of cancer-related death (1). NSCLC (Non-small cell lung cancer) is the most common type of lung cancer, and adenocarcinoma is the most common subtype of NSCLC (2, 3). Studies showed that most cancer patients die of cancer cell metastasis (4). In lung cancer, Lymph node metastasis is the most common way of metastasis (5). In the recent decades, SND (systematic nodal dissection), as a core method for evaluating node involvement levels at the mediastinal and hilar, has been accepted by the IASLC (International Association for Lung Cancer Research) as a key component of intrathoracic staging (6). However, for patients with no LN metastasis, SND has no other benefits except to prove that their pathological state is N0, which obviously leads to unnecessary invasive treatment. In addition, SND prevents the lymphatic fluid in the influenced area from being discharged, thereby resulting in lymphedema. This then leads to over-treatment. It is therefore important to develop a preoperative, non-invasive, and effective method to predict the extent of LN involvement.

Imaging methods, such as CT and PET (positron emission tomography), are commonly used in clinical LN diagnosis. CT can diagnose lymph nodes based on their size, but it cannot detect small LN metastasis. In PET imaging, LN metastasis usually shows increased FDG (Fludeoxyglucose) uptake, but inflammation and infection can also contribute to this. Compared with imaging methods, imaging-guided biopsy has better sensitivity and specificity in identifying LN metastasis, but it may lead to complications such as pneumothorax and bleeding (7–10). In recent years, radiomics has provided alternative ways for the diagnosis and prognosis of cancer (11–13). Some studies have successfully used radiomics features to predict LN metastasis in lung cancer (14, 15). In addition, thanks to the development of computer hardware and algorithms, deep learning has achieved great success in the field of computer vision (16). The model developed by deep learning has been successfully applied to the detection of skin cancer, diabetic retinopathy, breast cancer and so on (17–20). There are also studies related to deep learning in the diagnosis of lymph nodes of lung cancer (21, 22). However, few studies used both radiomics and deep learning to predict LN metastasis.

Therefore, the purpose of this study is to develop and validate the effectiveness of a nomogram (23) with a radiomics signature, deep learning signature, and clinical risk factors for the preoperative prediction of LN metastasis in patients with LUAD.

## Materials and Methods

### Patients and Data Acquisition

**Figure 1:**
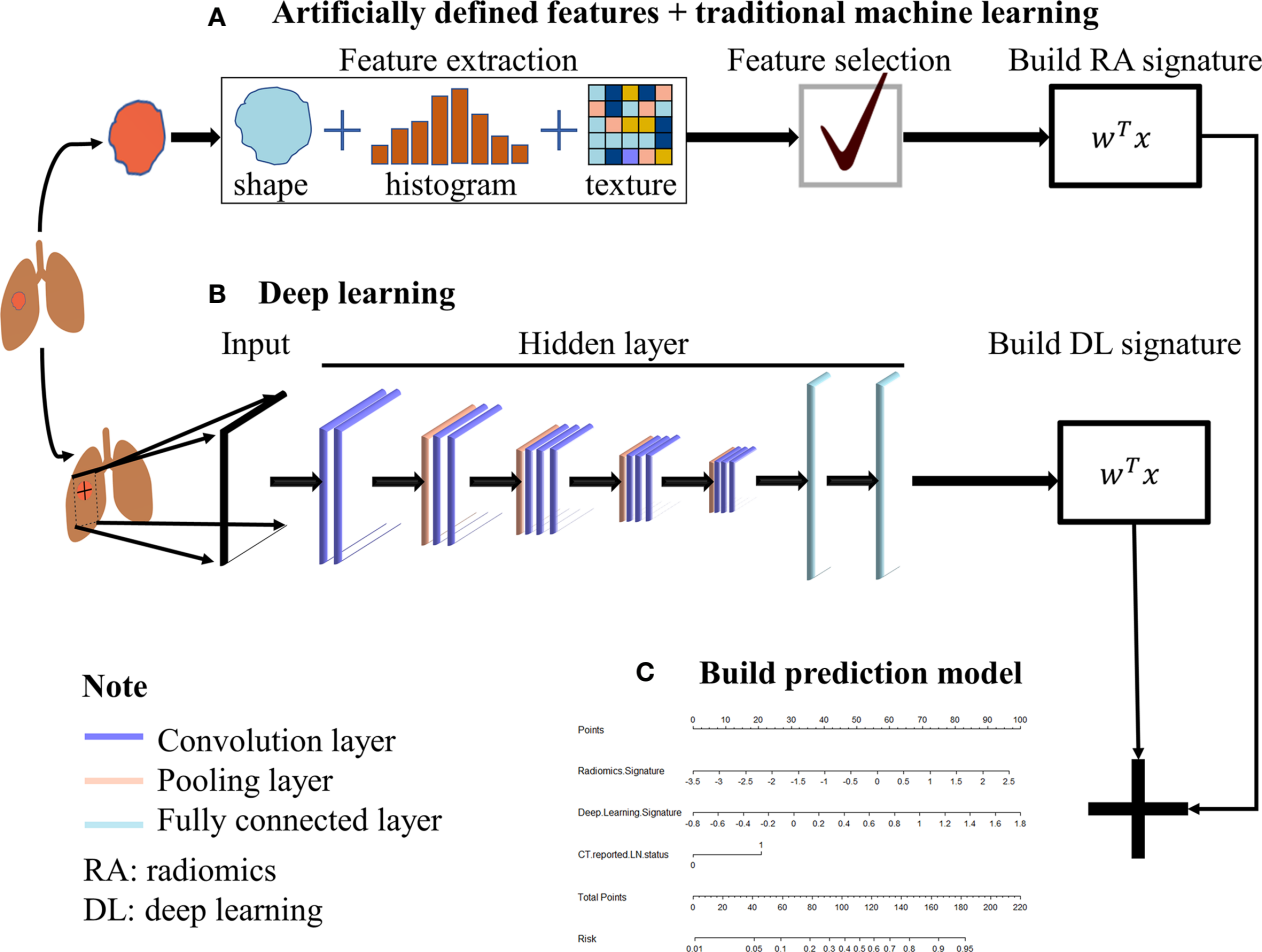
The workflow. **(A)** Traditional radiomics was used to extract artificial pre-defined features from the ROI region, and then the extracted features were selected and weighted to obtain the radiomics signature. **(B)** CNN was used to extract the automatic learning features from the slice where the ROI was located and then weighted to obtain deep learning signature. **(C)** Radiomics signature and deep learning signature were used to build the prediction model.

We retrospectively collected the data of 300 patients with LUAD from the Liaoning Cancer Hospital over the period of April 2015 to July 2019. We randomly divided 300 patients into the training cohort, internal validation cohort, and external validation cohort in equal proportions. In total, the training cohort included 200 patients: 99 males and 101 females; mean age, 63.21 ± 6.82. Internal validation cohort included 40 patients (18 males and 22 females; mean age, 64.35 ± 6.69). External validation cohort included 60 patients (27 males and 33 females; mean age, 63.18 ± 6.94). The baseline clinicopathological data included age, sex, CT-reported LN status, and CEA (carcinoembryonic antigen). However, owing to the lack of CEA data in more than half of the patients, CEA was abandoned. The inclusion and exclusion criteria of the data were as follows. Inclusion criteria: (a) the LN status was confirmed by operation and pathology reports, (b) the focus was single nodal mass type, (c) the time interval between CT scan and operation was no more than 1 month, (d) the slice thickness of CT plain scan image was 5 mm. Exclusion criteria: (a) preoperative radiotherapy or chemotherapy, (b) central lesions in the lung, (c) atelectasis and consolidation, (d) history of other tumors. The workflow of the study is illustrated in **Figure 1**.

Before CT scanning, foreign metal bodies were removed from the upper body of the patient to avoid the problem of artifacts. The patient was asked to raise his/her arms across the top of his/her head in the supine position and remain fixated in this position. The scanning was performed from the entrance of the chest to the diaphragm, when both the body and mind of the patient were in a relaxed state. The scanning machine used was Philips iCT 256 (Netherlands), and it had the following parameters: tube voltage of 120 kVp; 3D tube current in the range 110–325 mAs; layer thickness of 5.0 mm; acquisition matrix of 512 × 512; and, the FOV (field of view) was affected by the body fat and adjusted for thickness. The CT-reported LN status was determined by the radiologists based on the clinical radiological report of the preoperative CT. The presence of either regional LN of >1 cm and/or clusters of ≥3 lymph nodes was scored as LN-positive, and otherwise as LN-negative. The ROI (region of interest) was delineated by the radiologist according to the maximum cross-sectional area of the tumor boundary. CEA was obtained by a routine blood test and laboratory analysis within one week before operation. A CEA <5 ng/mL was recorded as normal, and otherwise as abnormal.

### Statistical Analysis

For determining the differences in the distribution of variables between cohorts, we used Kruskal-Wallis rank sum test to analyze the continuous variables (age, radiomics signature, deep learning signature) and chi-square test to analyze the discrete variables (sex, CT-reported LN status). Furthermore, for determining the correlation between variables and LN status within the cohort, we used Wilcoxon rank sum test to analyze the continuous variables and chi-square test or Fisher exact test to analyze the discrete variables. All the statistical tests in the study were two-sided with a significance level of 0.05.

### Building the Radiomics Signature and Deep Learning Signature

Pyradiomics (24) was used to extract features from the ROI. T-test was used to select features with a statistical significance of *P *<0.05, and Extra-trees was used to further select features with rich information from the training cohort. Then, logistic regression (25) was used to weight and combine the selected features, which built the radiomics signature. The extracted features and their weighting coefficients were applicable to the internal as well the external validation cohorts.

VGG-16 was used to build the deep learning signature. We first used each tumor slice and its adjacent two slices as R, G and B channels respectively, and combined them to obtain a three-channel image. Then an 80x80 pixel size area containing the tumor was cropped out as the final image input to VGG-16. As the amount of data collected was small, we used data augmentation to increase the amount of data and transfer learning to make the model easier to converge. Data augmentation technology included rotation, horizontal and vertical displacement, horizontal and vertical flipping, cropping, and scaling of the image. Transfer learning involved taking the pretraining weights of VGG-16 on the ImageNet dataset as the initial weights of the model, and then fine-tuning the model using the data of our training and internal validation cohorts. Next, the features of the last fully connected layer of VGG-16 were combined with weights and biases as the feature of each tumor image. The average value of the feature of multiple tumor images was calculated as the deep learning signature of the patient.

### Development of the Prediction Model

The candidate features of the multivariate logistic regression analysis included age, gender, CT-reported LN status, radiomics signature, and deep learning signature. The Akaike information criterion (26) was used as the stop criterion to determine the best features using a stepwise backward method. Additionally, the prediction model developed using logistic regression for the training cohort was also suitable for the internal as well as the external validation cohorts. Then, we developed a nomogram based on the developed prediction model.

### Performance of the Prediction model

The calibration curve and Hosmer-Lemeshow test (27) were used for model calibration. To quantify the discrimination, we calculated the C-index of the prediction model, and to compare the performance of the multi-factor model and the single-factor model, the NRI (net reclassification improvement) was calculated. In addition, we calculated the additional NRI of 5-fold cross-validation to obtain more reliable results.

Decision curve analysis (28) was used to quantify the net benefit at different threshold probabilities in the external validation cohort to determine the clinical value of the prediction model.

## Results

### Clinical Characteristics

The characteristics of all the cohorts are listed in **Table 1**. Because the data were divided into the different cohorts in equal proportions, the probability of LN metastasis was 50% in all the cohorts (*P*= 1.000). Furthermore, there was no significant difference observed with regard to gender among all the cohorts (*P*= 0.763), as was the case with the CT-reported LN status (*P*= 0.475) and age (*P*= 0.551). This indicated that the division of data was effective. Radiomics signature (*P*= 0.996) and deep learning signature (*P*= 0.869) also showed good reproducibility in all the cohorts (**Supplementary Material** and **Table S1**).

**Table 1:** **C**haracteristics of the patients in all the cohorts.

Characteristic	Training Cohort		*P*	Internal Validation Cohort		*P*	External Validation Cohort		*P*
	LN Metastasis (+)	LN Metastasis (–)		LN Metastasis (+)	LN Metastasis (-)		LN Metastasis (+)	LN Metastasis (-)	
Age, years			0.842			0.684			0.830
mean ± SD	62.97 ± 6.47	63.45 ± 7.19		64.75 ± 6.66	63.95 ± 6.88		63.00 ± 7.32	63.37 ± 6.67	
Gender, No. (%)			0.258			0.340			0.038^*^
Male	54(54.0)	45(45.0)		11(55.0)	7(35.0)		18(60.0)	9(30.0)	
Female	46(46.0)	55(55.0)		9(45.0)	13(65.0)		12(40.0)	21(70.0)	
CT-reported LN status, No. (%)			<0.001^*^			0.002^*^			0.010^*^
LN-negative	63(63.0)	85(85.0)		8(40.0)	18(90.0)		16(46.7)	26(86.7)	
LN-positive	37(37.0)	15(15.0)		12(60.0)	2(10.0)		14(53.3)	4(13.3)	
Radiomics signature,			<0.001^*^			<0.001^*^			<0.001^*^
median	0.529	-0.577		0.718	-0.657		0.731	-0.718	
(interquartile range)	(-0.034 to 1.120)	(-1.169 to 0.298)		(-0.023 to 1.035)	(-1.411 to -0.023)		(0.100 to 1.236)	(-1.376 to -0.250)	
Deep learning signature,			<0.001^*^			0.002^*^			<0.001^*^
median	0.670	0.227		0.667	0.121		0.770	0.199	
(interquartile range)	(0.440 to 1.002)	(-0.052 to 0.589)		(0.498 to 0.823)	(-0.095 to 0.479)		(0.421 to 1.165)	(-0.143 to 0.466)	

Univariate Analysis of Clinical Features and Lymph Node Status. Wilcoxon rank sum test was performed on continuous variables. Chi-square test was used for discrete variables whose theoretical frequency was greater than or equal to 5, and Fisher exact test was used for those less than 5.

*P value <.05.

### Building the Radiomics Signature and Deep Learning Signature

**Figure 2:**
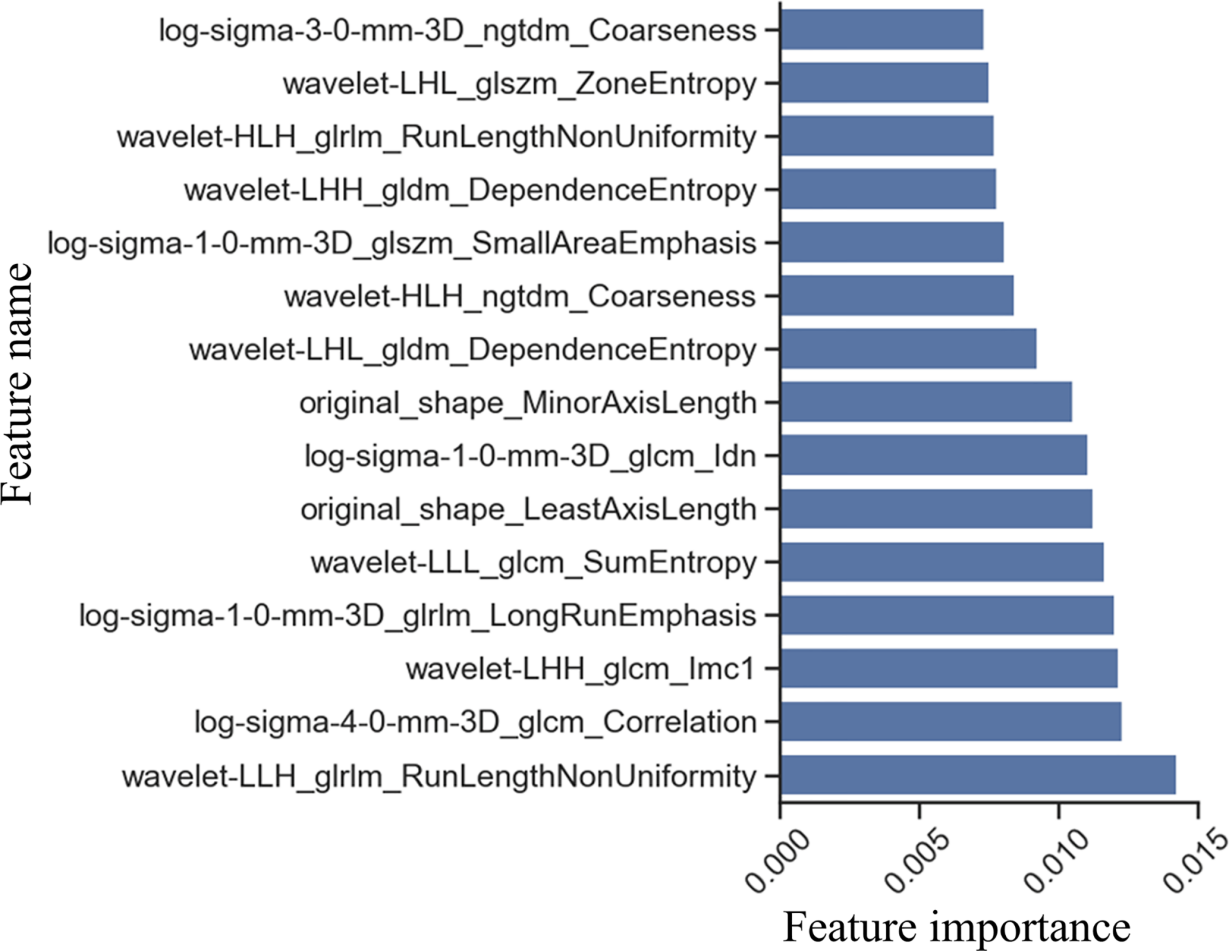
Feature selection by Extra-trees. Feature importance was obtained by averaging the results of multiple decision tree in Extra-trees. The larger the feature score, the more important is the feature.

A total of 1288 radiomics features were extracted from the CT images and the T-test was used to select 528 features (*P* <0.05) with good statistical significance. Next, Extra-trees was used to further select 8 features with rich information. The first eight features were selected because the eighth feature was a breakpoint, in other words, the value of the eighth feature was significantly different from that of the ninth feature. After the ninth feature, the importance of the features changed slightly (**Figure 2**). For the eight selected features, we used logistic regression for performing weighted summation to obtain the radiomics signature. The distribution of the radiomics signature showed that the signature had good separability in metastasis and not in the metastasis categories (**Table 1**).

**Figure 3:**
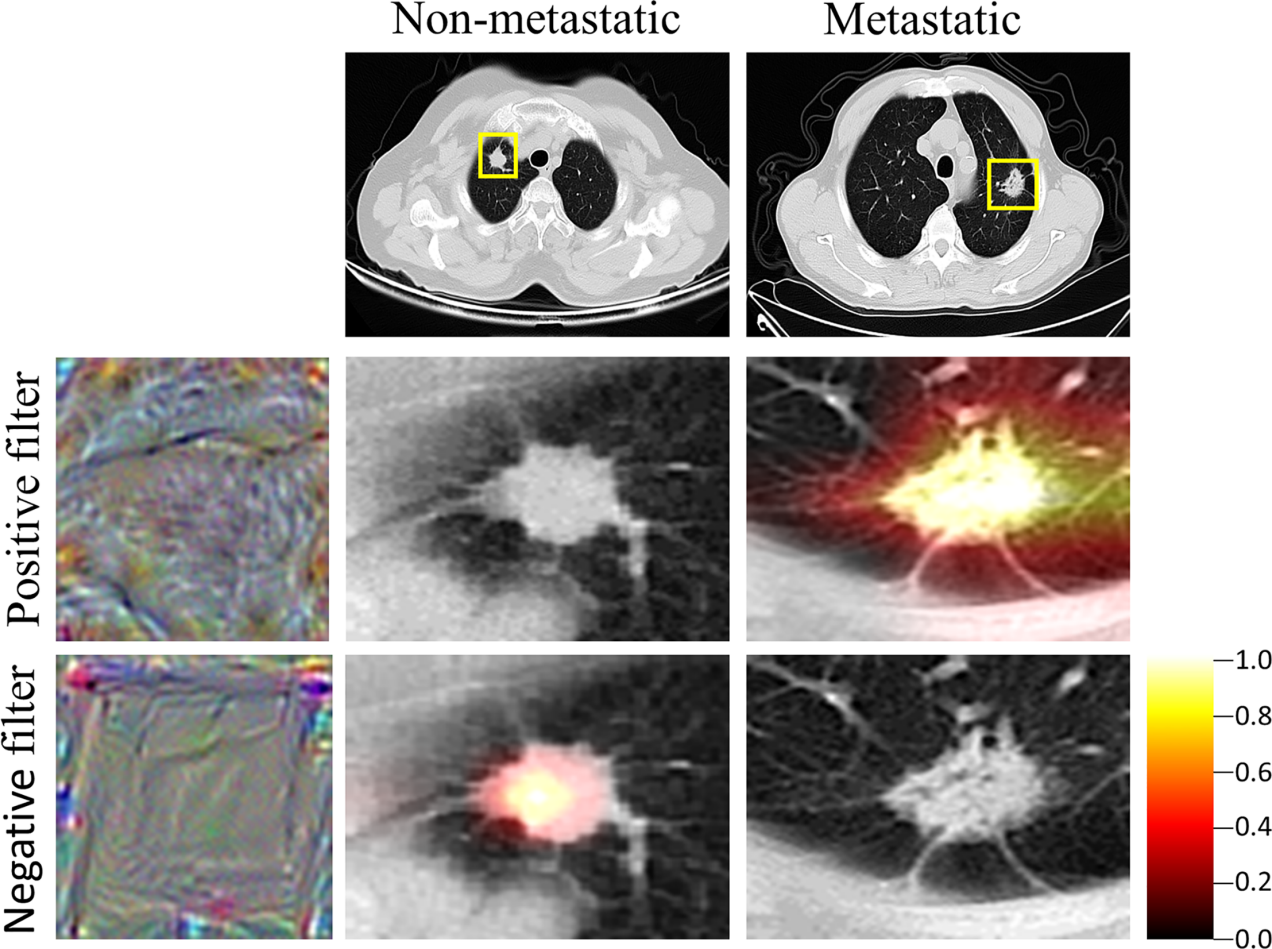
Heat map of VGG. Grad-cam showed the region of ​​interest of the VGG, which was also the region where the features were extracted by the VGG. The closer the color is to yellow, the more important is the region.

The features of the last fully connected layer of VGG-16 were weighted to obtain the deep learning signature. To help users build trust in VGG-16 predictions, the grad-cam (29) method was used to generate a heat map. The heat map tells the user the position of the feature on which the prediction is based in the image, and uses the color depth to represent the importance of the feature. The deeper the color is, the more important the feature in the region is. In this study, the heat maps of two of the filters in the last convolution layer of VGG-16 were plotted. The heat maps suggested that the positive filter focused on the features of metastatic LN, ignoring the features of non-metastatic LN, while the negative filter focused on the features of non-metastatic LN, thus ignoring the features of metastatic LN (**Figure 3**). This indicated that the LN features extracted by VGG-16 can distinguish LN metastasis from non-metastatic, and the distribution of the deep learning signature further confirmed this finding (**Table 1**).

### Development of the Prediction Model

**Figure 4:**
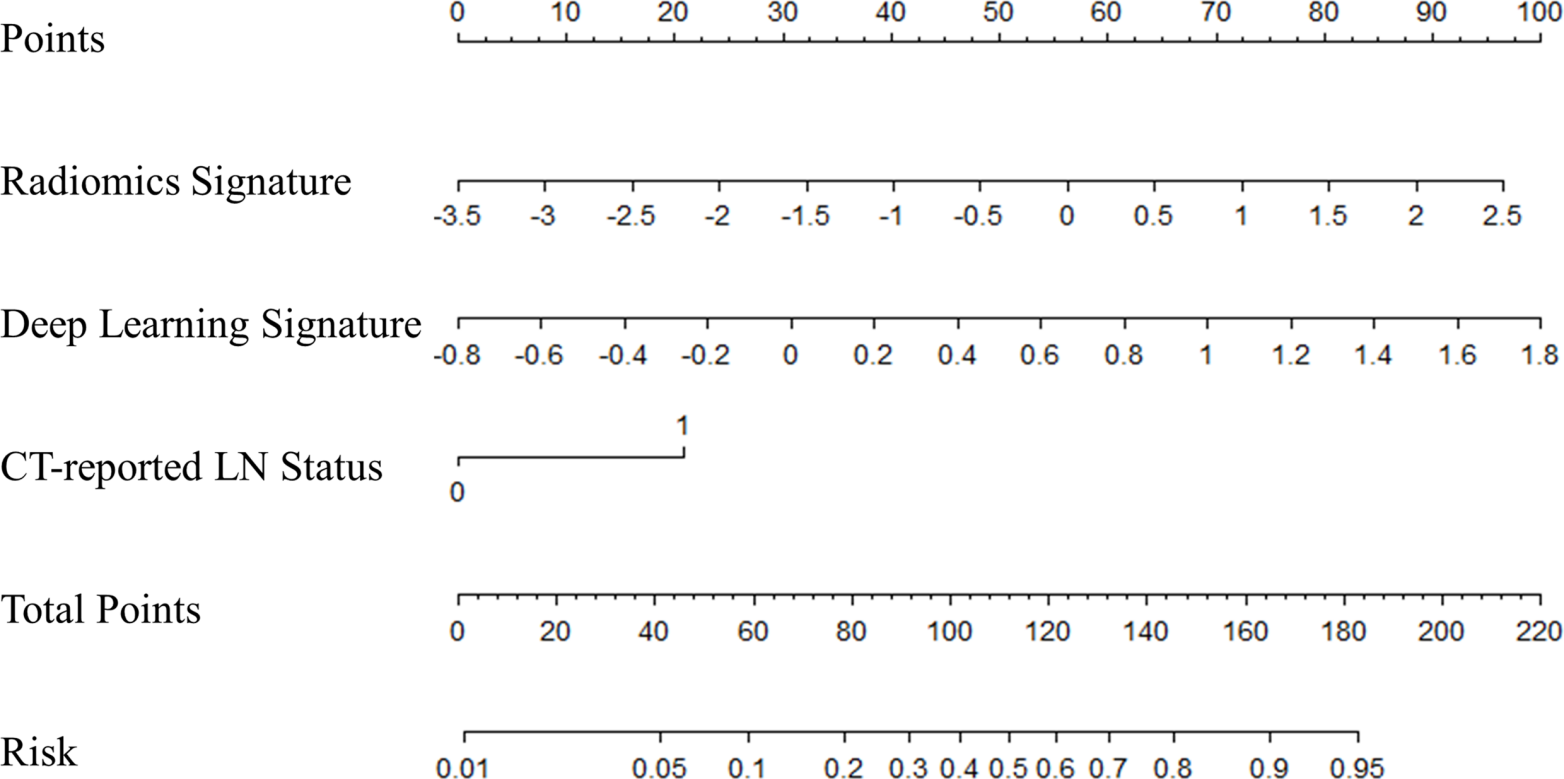
The nomogram of multifactor model. Nomogram was built for the training cohort, using the radiomics signature, deep learning signature, and CT-reported LN status.

Multivariate logistic regression analysis confirmed that radiomics signature, deep learning signature, and CT-reported LN status were independent predictors (**Table 2**). The model combining the above-mentioned independent predictors was developed and presented in the form of a nomogram (**Figure 4**). The specific method for estimating the LN metastasis probability is explained in the **Supplementary Material (Equation S1)**.

**Table 2:** Risk factors for LN metastasis in LUAD.

Intercept and Variable	Model 1		*P*	Model 2		*P*	Model 3		*P*	Model 4		*P*
	β	Odds Ratio (95% CI)		β	Odds Ratio (95% CI)		β	Odds Ratio (95% CI)		β	Odds Ratio (95% CI)	
Intercept	-0.004		0.982	-1.211		<0.001	-0.300		0.072	-1.031		0.001
Radiomics signature	1.088	2.967 (2.122 to 4.327)	<0.001	NA			NA			0.669	1.951 (1.299 to 3.010)	0.002
Deep learning signature	NA			2.454	11.630 (5.457 to 27.288)	<0.001	NA			1.598	4.944 (1.968 to 13.239)	0.001
CT-reported LN status	NA			NA			1.202	3.328 (1.710 to 6.748)	<0.001	0.868	2.383 (1.127 to 5.194)	0.025
C-index												
Training cohort	0.775(0.712 to 0.838)			0.777(0.713 to 0.841)			0.610(0.551 to 0.669)			0.820(0.762 to 0.879)		
Internal validation cohort	0.810(0.674 to 0.946)			0.785(0.627 to 0.943)			0.750(0.624 to 0.876)			0.830(0.694 to 0.966)		
external validation cohort	0.844(0.744 to 0.945)			0.812(0.705 to 0.919)			0.667(0.559 to 0.775)			0.861(0.769 to 0.954)		

β is the coefficient of logistic regression. Odds ratio means the rate of change of occurrence of the event when the other factors remain unchanged and this factor changes by a single unit. NA means not available.

### Performance of the Prediction Model

**Figure 5:**
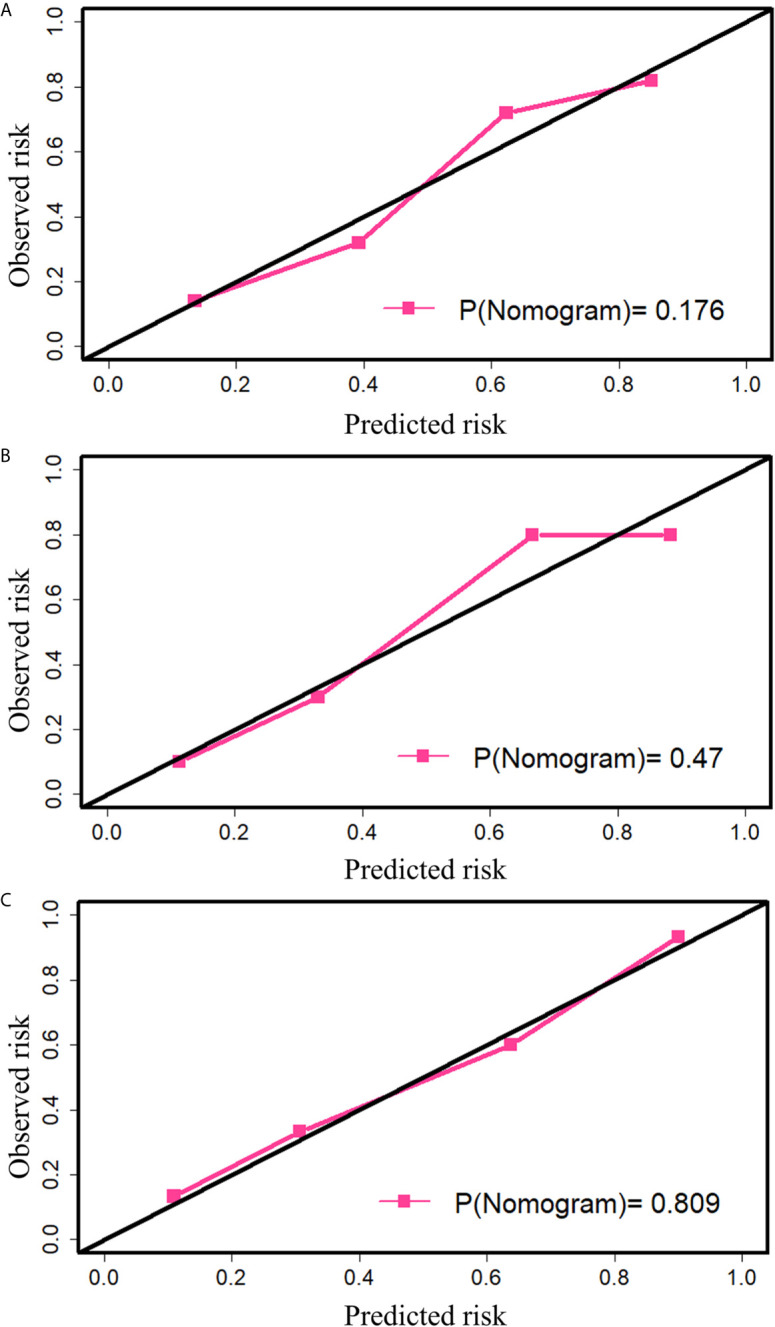
The calibration plot of nomogram. **(A)** Calibration in the training cohort. **(B)** Calibration in the internal validation cohort. **(C)** Calibration in the external validation cohort. The black diagonal indicates the ideal fit, and in this case, the metastasis probability predicted by the nomogram is the same as the actual observed transition probability. The pink line indicates the fitting of the nomogram. The closer it is to the black line, the better is the fit. P-value was calculated by the Hosmer-Lemeshow test.

The calibration curve (**Figure 5**) of the nomogram showed that there was a good agreement between the prediction and observation results in the training cohort (*P*= 0.193), indicating no deviation from the perfect fit. The nomogram also showed good agreement in the internal (*P*= 0.468) as well as the external (*P*= 0.824) validation cohorts. Furthermore, it showed good discrimination capability for all the cohorts, and the C-index of nomogram in the training cohort, internal validation cohort and external validation cohort was 0.820 (95% CI, 0.762 to 0.879), 0.830 (95% CI, 0.694 to 0.966) and 0.861 (95% CI, 0.769 to 0.954) respectively.

To measure the improvement of the multi-factor nomogram over other single-factor prediction models, the NRI was calculated for the external validation cohort. Based on the results, it was confirmed that the nomogram showed a significant improvement compared with the model built using only radiomics signature (IDI, 0.087; *P*= 0.022; NRI (Categorical), 0.033 and NRI (Continuous), 0.667), only deep learning signature (IDI, 0.101; *P *<0.001; NRI (Categorical), 0.133 and NRI (Continuous), 1.133), and only CT-reported LN status (IDI, 0.297; *P *<0.001; NRI (Categorical), 0.267 and NRI (Continuous), 1.067). The NRI results of an additional 5-fold cross-validation also showed that the multi-factor model was significantly improved compared to the single-factor model (**Supplementary Material, Table S2**). This proved that the deep learning signature was helpful for improving the performance of the prediction model.

**Figure 6:**
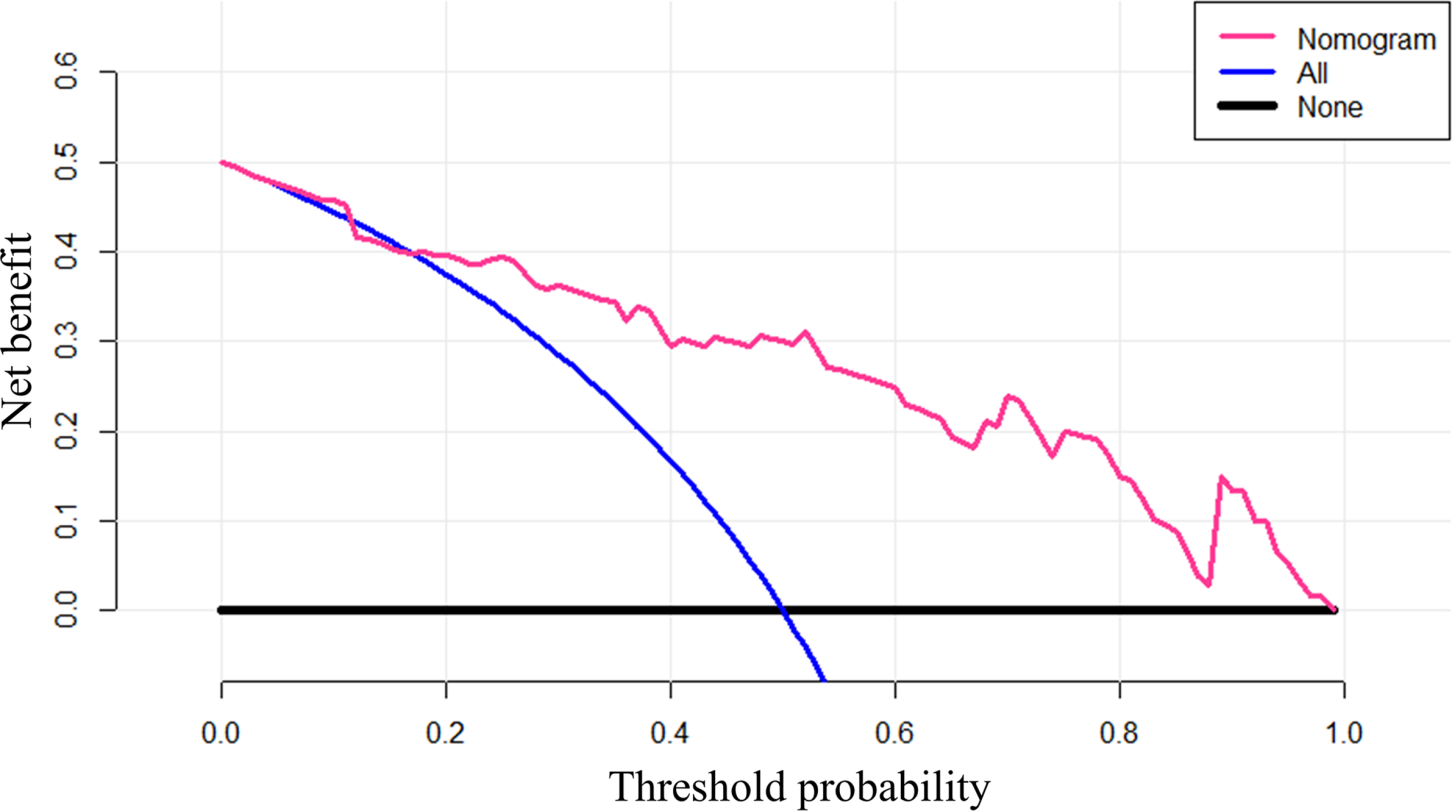
The decision curve of nomogram. Net benefit was calculated as $Net\;Benefit\;=\;\frac{True\;Positive}{n}\;-\;\frac{False\;Positive}{n}\;(\frac{Pt}{1-Pt})$, where n is the total number of patients, true positive is the number of patients predicted by the model as positive and actually positive, and false positive is the number of patients predicted as positive and actually negative. *p_t_* is the threshold probability.

The decision curve showed that if the threshold probability of determining the presence of LN metastasis was greater than 0.18, using nomogram to predict LN metastasis will benefit more than the all the treatment plans or no treatment plan at all (**Figure 6**).

## Discussion

In this study, we developed and validated a nomogram based on a radiomics signature, deep learning signature, and CT-reported LN status for the preoperative prediction of LN metastasis in patients with LUAD.

For constructing the radiomics signature, the total number of features (1288) was reduced to 528 using the T-test, and then 8 features with rich information were selected using Extra-trees. Extra-trees is an ensemble learning method, which is composed of multiple decision tree. Extra-trees reduces the risk of overfitting a single model. Therefore, the features selected by Extra-trees are robust. Then, we used logistic regression to combine features to obtain the radiomics signature.

Considering that deep learning achieves better results than traditional machine learning methods in ImageNet large-scale Visual recognition Challenge, this study also used deep learning method. The difference between radiomics method and deep learning method is that the extracted features are different. The features extracted by the deep learning method are automatically learned by the model from the data, while the features extracted by the radiomics method are artificially defined. These two approaches have their own advantages and disadvantages. For the deep learning method, if the amount of data is large enough, the features it learns can explain the data well, but when the amount of data is small, the features it learns can’t explain the data well. For the radiomics method, the features extracted by it are not affected by the amount of data, but by artificial experience. Limited artificial experience makes it difficult for radiomics method to achieve very good diagnostic results. The combination of radiomics features and deep learning features can integrate the advantages of both. Therefore, in this study, both deep learning features and radiomics features were used to predict LN metastasis of lung adenocarcinoma. However, different from some studies, we didn’t directly output thousands of features of the last fully connected layer, but combined the features with its weights and biases to get a deep learning signature (30, 31). This helped to draw nomogram and analyze the individual influence of deep learning features on LN metastasis.

Because the amount of data is relatively small, this study used data augmentation technology and transfer learning technology in deep learning. Data augmentation technology expanded the amount of data. Transfer learning technology made the training of VGG-16 easier. Specifically, we took the pretraining weights of VGG-16 on ImageNet as the initial weights of the model, and then used our data to fine-tune the model.

Multivariate logistic regression analysis showed that radiomics signature, deep learning signature and CT-reported LN status were independent and effective predictors. The c-index of the nomogram constructed with these three features in the training cohort, the internal validation cohort and the external validation cohort was respectively 0.820 (95% CI, 0.762 to 0.879), 0.830 (95% CI, 0.694 to 0.996), 0.861 (95% CI, 0.769 to 0.954), which was better than any single-factor model. The results of NRI showed that nomogram was significantly improved compared with the single-factor model.

The limitations of this study mainly include the following: (a) No enough clinical information. Smoking history and CEA have been proved to be effective predictors of LN metastasis (14, 15); (b) No genetic information was used. Some studies have shown that in the primary tumor, miR-31, miR-34b/c, miR-148 and miR-9-325 were significantly correlated with LN status (32, 33). Incorporating genetic features may improve the performance of the radiomics model, which may be a future research direction; (c) The amount of data is relatively small. The more the amount of data, the features learned by the deep learning method can better explain the data; (d) This is a single-center retrospective study. A prospective multicenter clinical trial is needed to validate our model.

In summary, this study proposes a nomogram based on radiomics signature, deep learning signature, and CT-reported LN status that can be conveniently used to predict preoperative LN metastasis in patients with LUAD.

## Data Availability Statement

The data analyzed in this study is subject to the following licenses/restrictions: The datasets are privately owned by Liaoning Cancer Hospital and are not made public. Requests to access these datasets should be directed to DZ, zhaodan777@126.com.

## Ethics Statement

The studies involving human participants were reviewed and approved by Medical Ethics Committee of Cancer Hospital of Liaoning Province, China. Written informed consent for participation was not required for this study in accordance with the national legislation and the institutional requirements.

## Author Contributions

JR, RC, JC, DZ, and ZW conceived and designed the concept. JC, TY, and DZ collected the data. JR and RC analyzed the data. All authors contributed to the article and approved the submitted version.

## Funding

This study was supported by the National Key Research and Development Program of China (Nos. 2017YFC1309100), and the Science and Technology Program of Liaoning Province (No. 2018225038).

## Conflict of Interest

The authors declare that the research was conducted in the absence of any commercial or financial relationships that could be construed as a potential conflict of interest.

## Abbreviations

CNN, Convolutional neural network; CT, computed tomography; FDG, Fludeoxyglucose; FOV, field of view; IASLC, International Association for Lung Cancer Research; LUAD, lung adenocarcinoma; LN, lymph node; NRI, net reclassification improvement; PET, positron emission tomography; ROI, region of interest.
