# Supplementary material for: Development and Validation of a Nomogram for Preoperative Prediction of Lymph Node Metastasis in Lung Adenocarcinoma Based on Radiomics Signature and Deep Learning Signature
Source: Front Oncol. 2021 Apr 22;11:585942. doi: 10.3389/fonc.2021.585942 (PMC8101496; doi:10.3389/fonc.2021.585942)
Supplement: Supplementary file 1 [file DataSheet_1.docx]

| **Table S1 \|** Rationality of data division | | | | |
| --- | --- | --- | --- | --- |
| Characteristic | Training Cohort | Internal Validation Cohort | External Validation Cohort | *P* |
| Age  mean±sd | 63.21±6.82 | 64.35±6.69 | 62.18±6.94 | 0.551 |
| Gender  male/female | 99/101 | 18/22 | 27/33 | 0.763 |
| CT-reported LN status  LN-negative  LN-positive | 148  52 | 26  14 | 42  18 | 0.475 |
| Radiomics signature  median (interquartile range) | 0.036(-0.791 to 0.740) | -0.025(-0.822 to 0.873) | -0.197(-0.898 to 0.790) | 0.996 |
| Deep learning signature  median (interquartile range) | 0.494(0.148 to 0.846) | 0.481(0.080 to 0.771) | 0.434(0.080 to 0.867) | 0.869 |

Chi-square test for discrete variables, and Kruskal-Wallis Rank Sum Test for continuous variables.

| **Table S2 \|** NRI of 5-fold cross-validated | | | | | | | |
| --- | --- | --- | --- | --- | --- | --- | --- |
|  |  | Model1 and Model4 | | Model2 and Model4 | | Model3 and Model4 | |
| k |  |  | *P* |  | *P* |  | *P* |
| 1 | NRI(Categorical, 95% CI) | 0.069 | 0.498 | -0.042 | 0.679 | 0.444 | 0.000 |
|  | NRI(Continuous, 95% CI) | 0.444 | 0.067 | 0.694 | 0.005 | 1.222 | 0.000 |
|  | IDI (95% CI) | 0.062 | 0.046 | 0.057 | 0.154 | 0.258 | 0.000 |
| 2 | NRI(Categorical, 95% CI) | 0.039 | 0.731 | 0.156 | 0.185 | 0.075 | 0.524 |
|  | NRI(Continuous, 95% CI) | 0.615 | 0.008 | 0.896 | 0.000 | 0.968 | 0.000 |
|  | IDI (95% CI) | 0.066 | 0.047 | 0.133 | 0.001 | 0.226 | 0.000 |
| 3 | NRI(Categorical, 95% CI) | 0.067 | 0.568 | 0.000 | 1.000 | 0.058 | 0.658 |
|  | NRI(Continuous, 95% CI) | 0.580 | 0.018 | 0.795 | 0.001 | 0.848 | 0.000 |
|  | IDI (95% CI) | 0.061 | 0.094 | 0.092 | 0.000 | 0.199 | 0.001 |
| 4 | NRI(Categorical, 95% CI) | 0.223 | 0.013 | 0.040 | 0.739 | 0.337 | 0.006 |
|  | NRI(Continuous, 95% CI) | 0.469 | 0.062 | 0.297 | 0.250 | 0.834 | 0.000 |
|  | IDI (95% CI) | 0.062 | 0.030 | 0.040 | 0.403 | 0.245 | 0.000 |
| 5 | NRI(Categorical, 95% CI) | 0.206 | 0.070 | 0.291 | 0.011 | 0.291 | 0.003 |
|  | NRI(Continuous, 95% CI) | 0.834 | 0.000 | 0.754 | 0.002 | 0.766 | 0.002 |
|  | IDI (95% CI) | 0.089 | 0.011 | 0.099 | 0.002 | 0.229 | 0.000 |

Model1: radiomics signature

Model2: deep learning signature

Model3: CT-reported LN status

Model4: radiomics signature + deep learning signature + CT-reported LN status

$$probability=\frac{1}{1+e^{-(wx+b)}}$$

$$wx+b=0.669*Radiomics signature+1.598*Deep learning signature+0.868*CT\_reported LN status-1.031$$

**Equation S1 |** Calculation of LN metastasis probability
